# Supplementary material for: Prognostic role of PD-L1 expression in patients with salivary gland carcinoma: A systematic review and meta-analysis
Source: PLoS One. 2022 Jul 26;17(7):e0272080. doi: 10.1371/journal.pone.0272080 (PMC9321421; doi:10.1371/journal.pone.0272080)
Supplement: S1 Table — (DOCX) [file pone.0272080.s001.docx]

S1 Table. Search strategy of database

| **Database** | **Search strategy** |
| --- | --- |
| **PubMed** | (((((((survival)[Title/Abstract] OR (prognostic))[Title/Abstract] OR (prognosis))[Title/Abstract] OR (outcome))[Title/Abstract] OR (mortality))[Title/Abstract] AND ((((tumor)[Title/Abstract] OR (neoplasm))[Title/Abstract] OR (cancer))[Title/Abstract] OR (carcinoma)))[Title/Abstract] AND (((((((salivary gland)[Title/Abstract] OR (parotid gland))[Title/Abstract] OR (submandibular gland))[Title/Abstract] OR (sublingual gland))[Title/Abstract] OR (salivary duct))[Title/Abstract] OR (adenoid cystic))[Title/Abstract] OR (mucoepidermoid)))[Title/Abstract] AND ((((PD-L1)[Title/Abstract] OR (B7-H1))[Title/Abstract] OR (CD274))[Title/Abstract] OR (programmed cell death ligand 1))[Title/Abstract] |
| **Scopus** | TITLE-ABS-KEY ( "PD-L1"  OR  "B7-H1"  OR  "CD274"  OR  "programmed cell death ligand 1" )  AND TITLE-ABS-KEY ( " salivary gland "  OR  " parotid gland "  OR  " submandibular gland "  OR  " sublingual gland "  OR " salivary duct" OR  " adenoid cystic " OR  " mucoepidermoid " )  AND TITLE-ABS-KEY ( " tumor "  OR  " neoplasm "  OR  " cancer "  OR  " carcinoma " )  AND TITLE-ABS-KEY ( " survival "  OR  " prognostic "  OR  " prognosis "  OR  " outcome " OR  " mortality " ) |
| **Embase** | ('pd-l1':ab,ti OR 'b7-h1':ab,ti OR 'cd274':ab,ti OR 'programmed cell death ligand 1':ab,ti) AND ('salivary gland':ab,ti OR 'parotid gland':ab,ti OR 'submandibular gland':ab,ti OR 'sublingual gland':ab,ti OR 'salivary duct':ab,ti OR 'adenoid cystic':ab,ti OR 'mucoepidermoid':ab,ti) AND ('tumor':ab,ti OR 'neoplasm':ab,ti OR 'cancer':ab,ti OR 'carcinoma':ab,ti) AND ('survival':ab,ti OR 'prognostic':ab,ti OR 'prognosis':ab,ti OR 'outcome':ab,ti OR 'mortality':ab,ti) |
