# Supplementary material for: Prognostic role of PD-L1 expression in patients with salivary gland carcinoma: A systematic review and meta-analysis
Source: PLoS One. 2022 Jul 26;17(7):e0272080. doi: 10.1371/journal.pone.0272080 (PMC9321421; doi:10.1371/journal.pone.0272080)
Supplement: S1 Fig — (DOCX) [file pone.0272080.s005.docx]

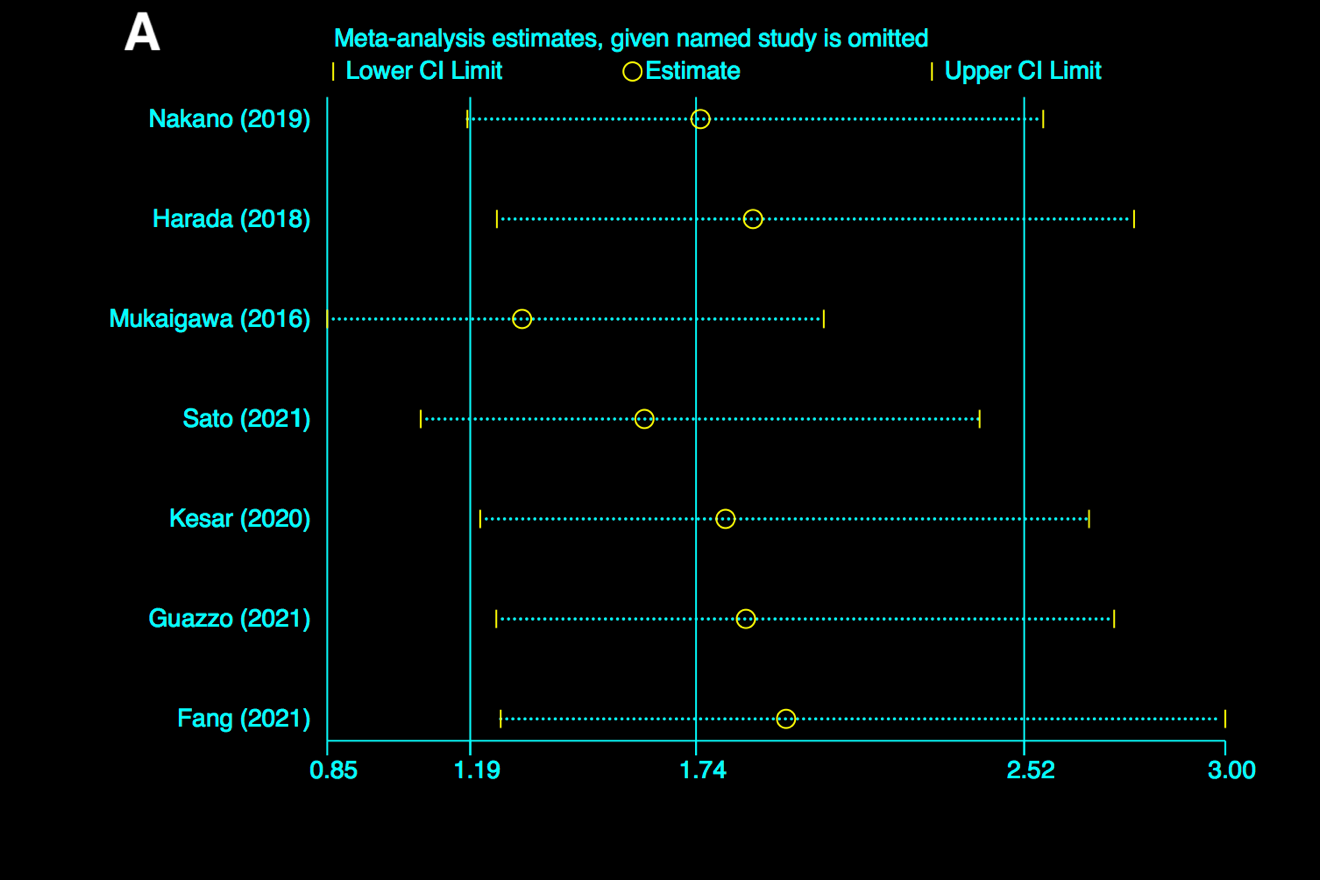


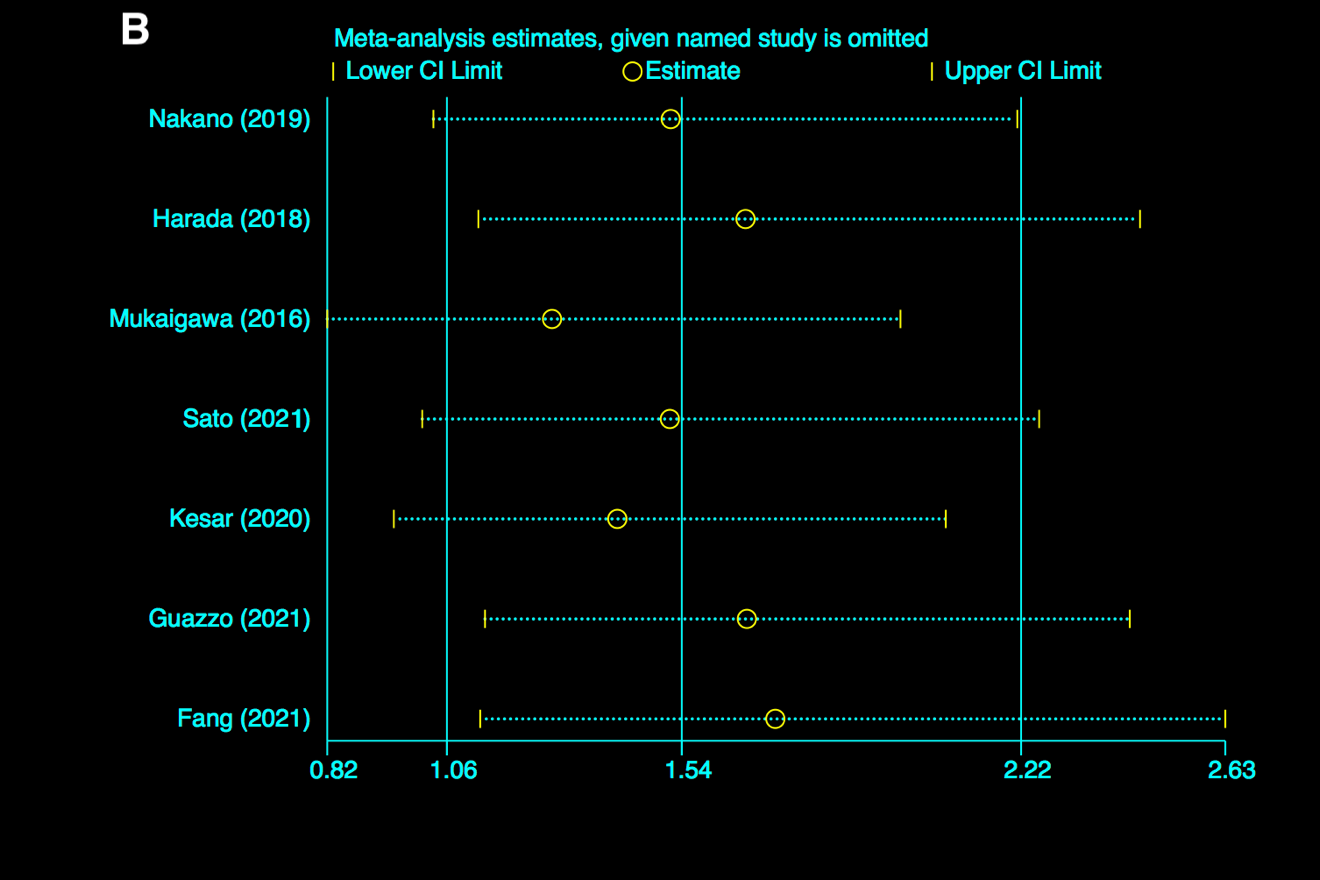


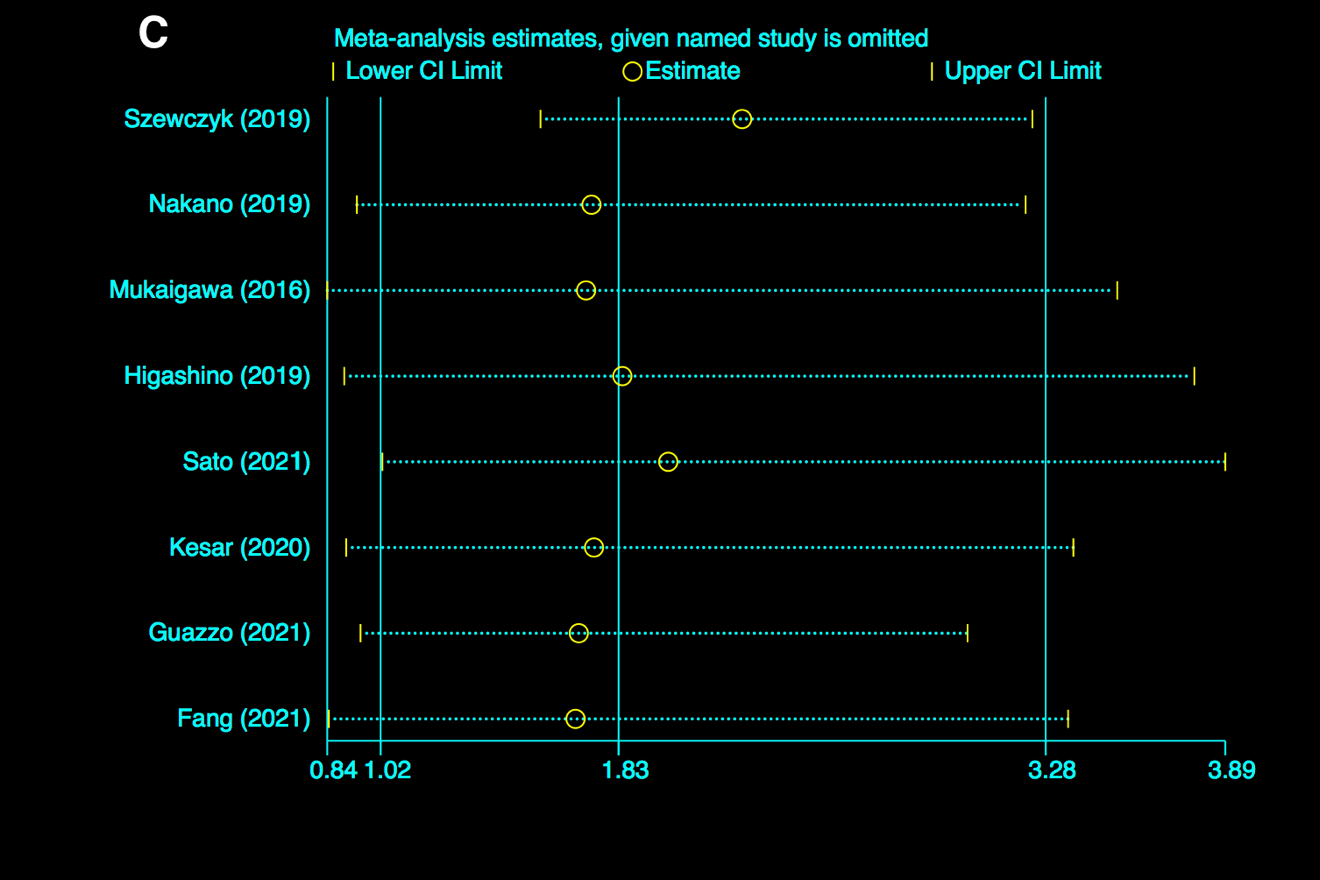


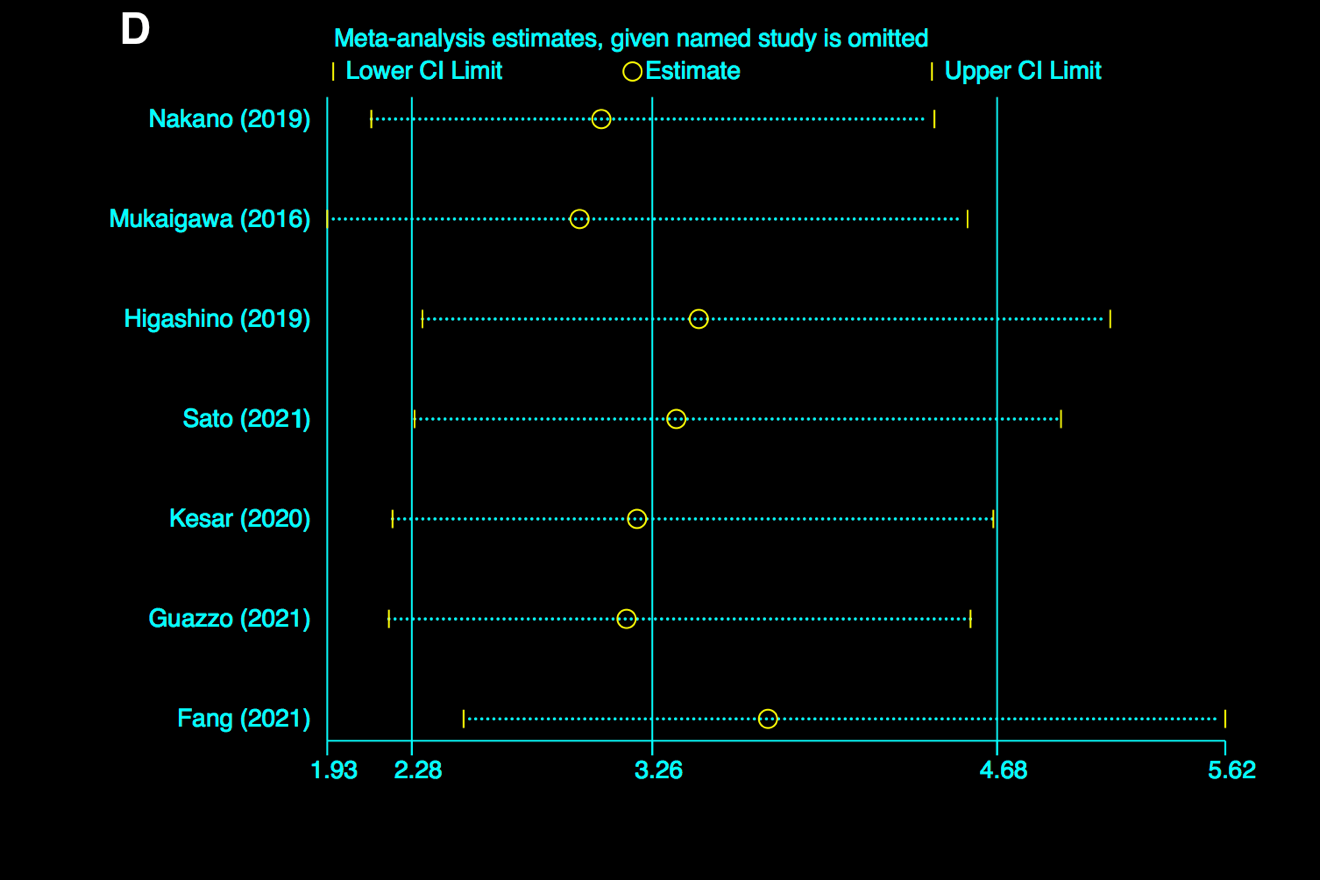


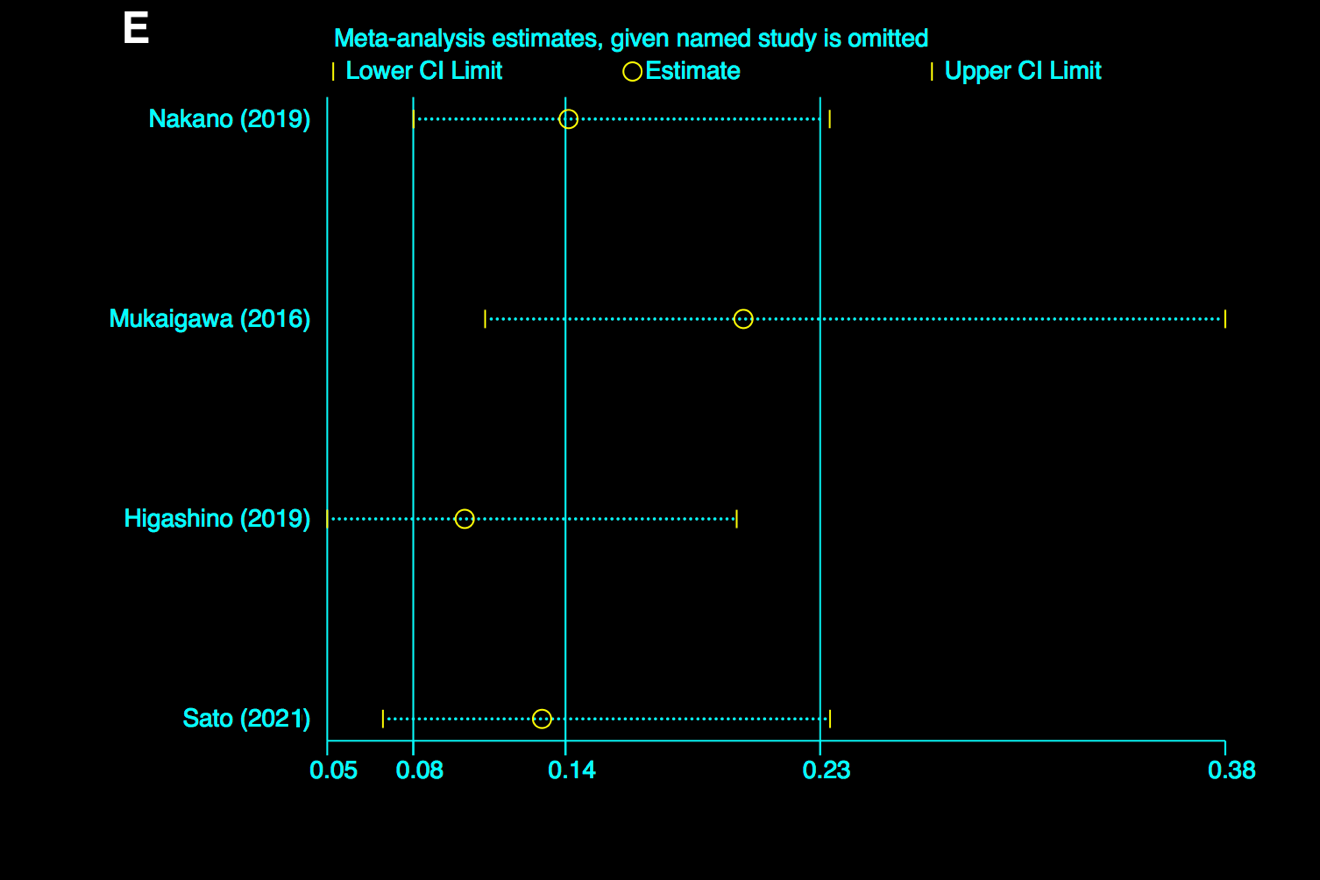


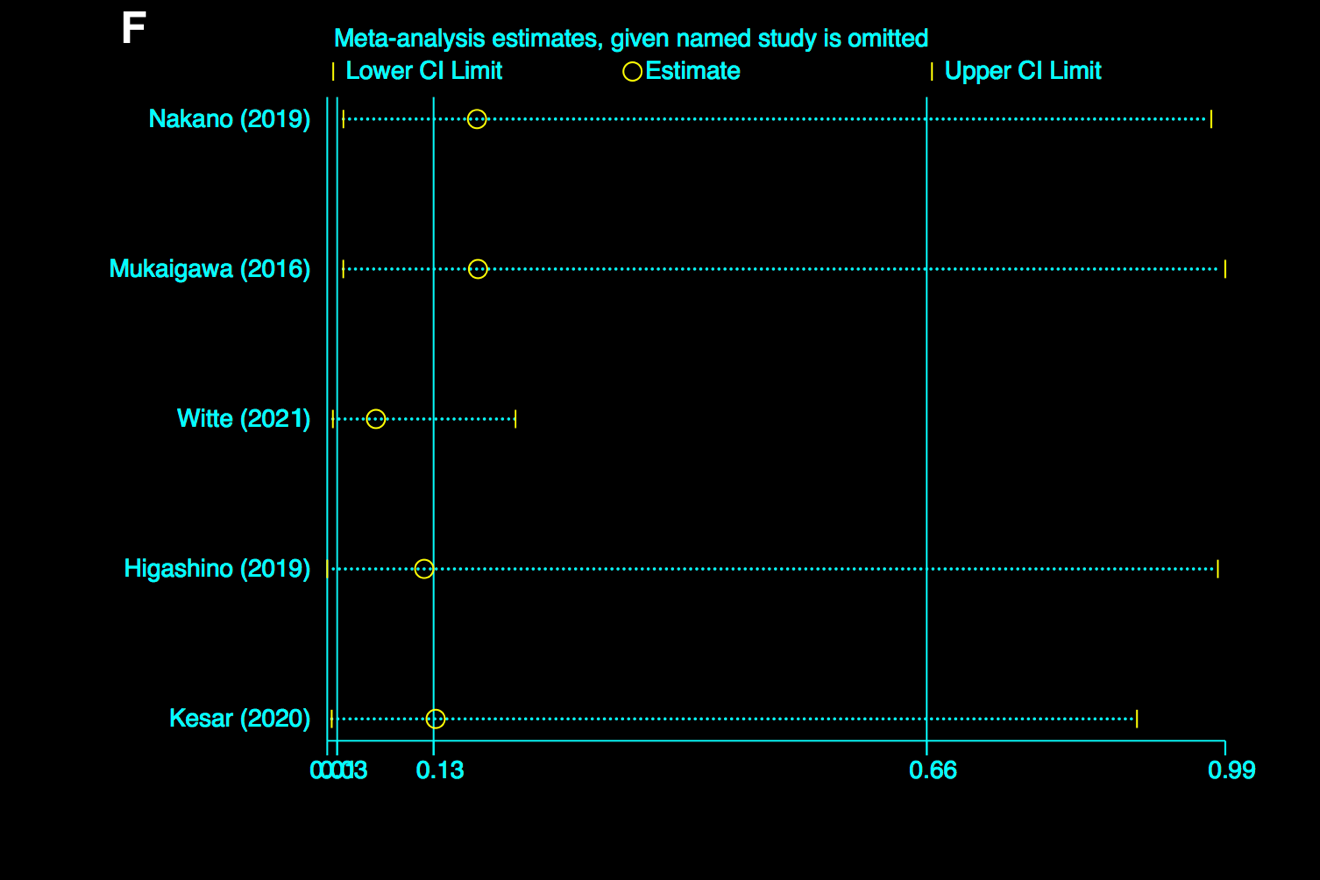


S1 Fig. Sensitivity analysis in (A) Gender, (B)Age, (C) T Stage, (D) N Stage, (E) Histological diﬀerentiation, (F) Subtype (non-ACC&ACC).


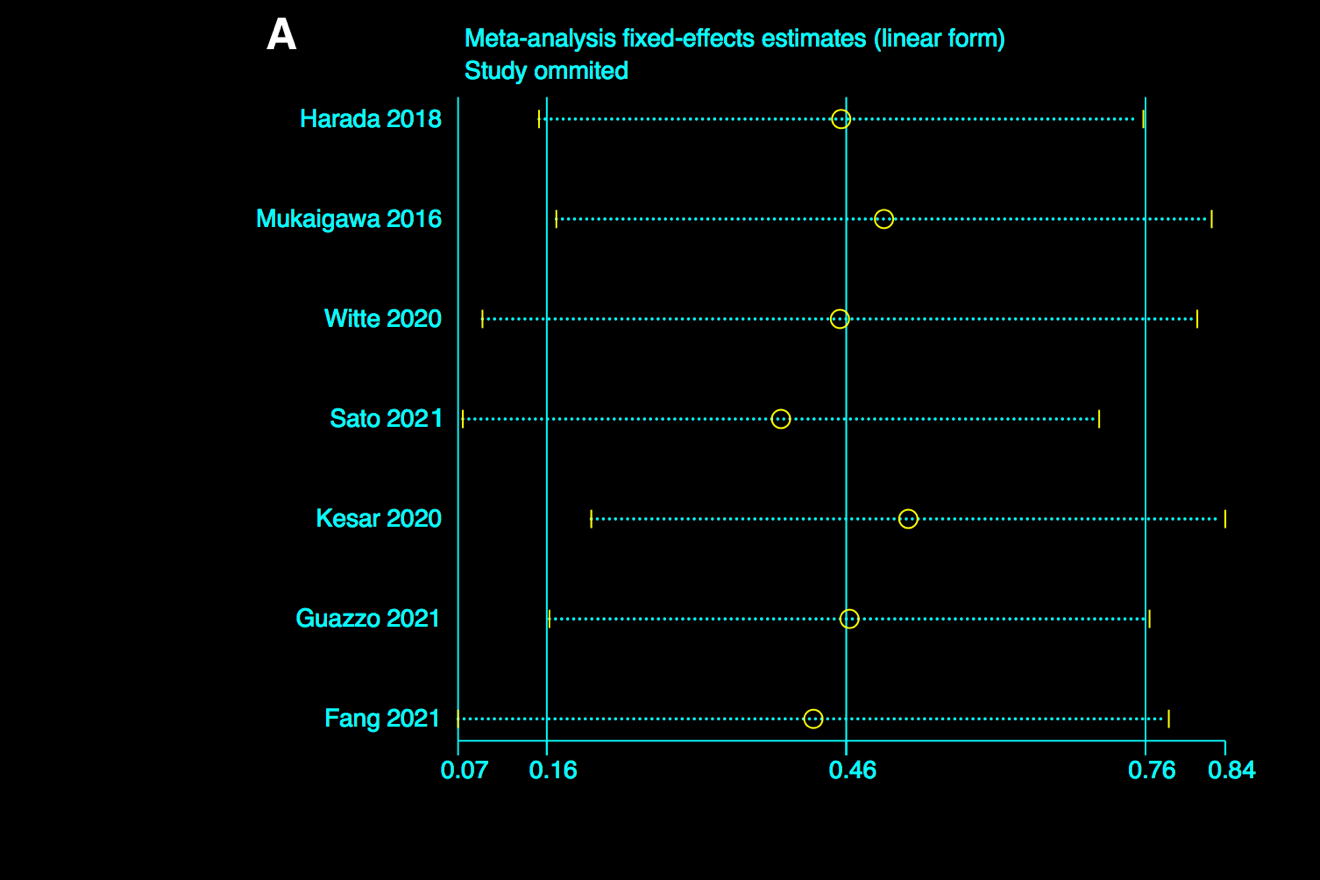


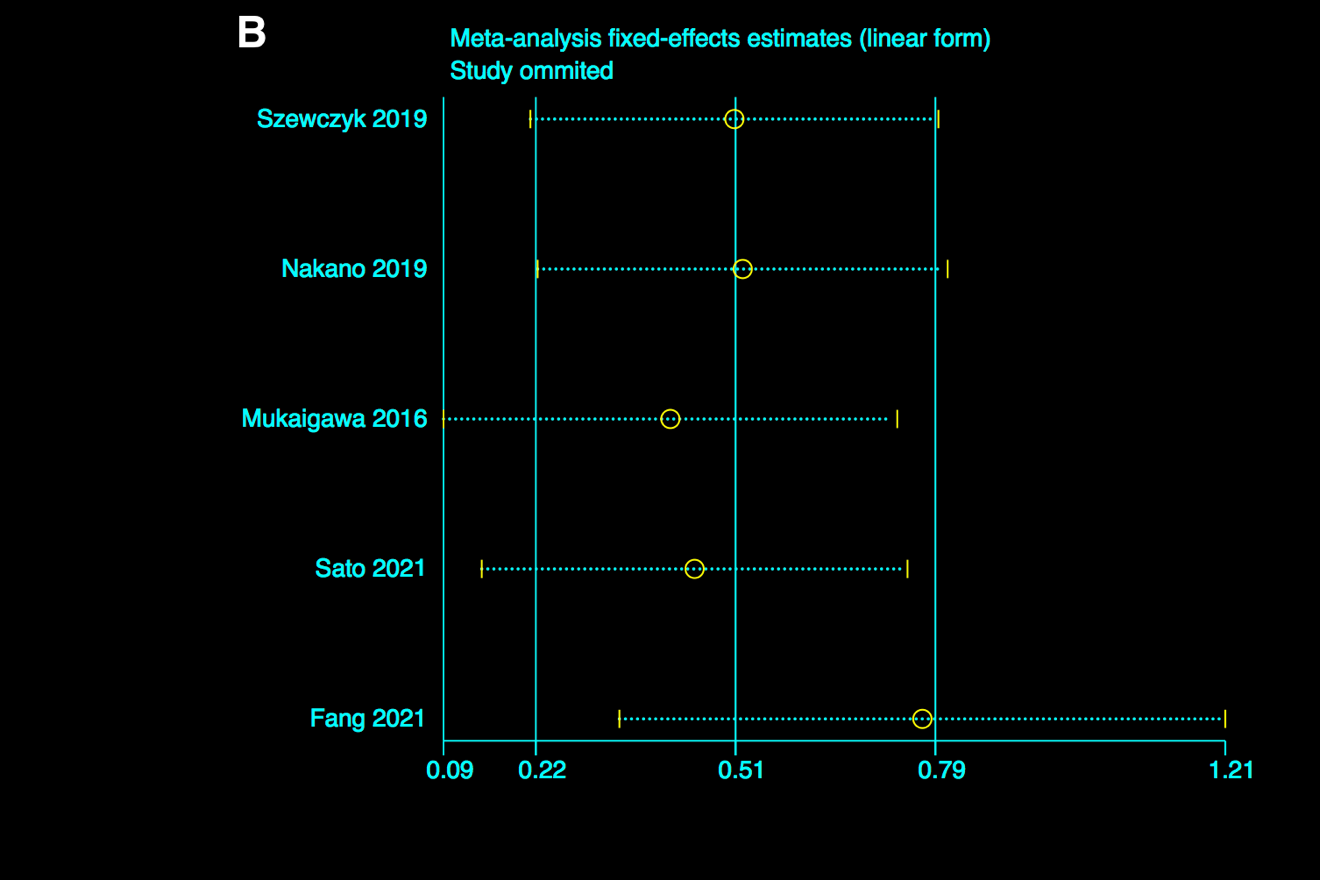


S2 Fig. Sensitivity analysis of the relationship between PD-L1 expression and (A) OS, (B) DFS.
